# Migratory goose arrival time plays a larger role in influencing forage quality than advancing springs in an Arctic coastal wetland

**DOI:** 10.1371/journal.pone.0213037

**Published:** 2019-03-13

**Authors:** Karen H. Beard, Ryan T. Choi, A. Joshua Leffler, Lindsay G. Carlson, Katharine C. Kelsey, Joel A. Schmutz, Jeffrey M. Welker

**Affiliations:** 1 Department of Wildland Resources, Utah State University and the Ecology Center, Logan, UT, United States of America; 2 Department of Natural Resource Management, South Dakota State University, Brookings, SD, United States of America; 3 Department of Biological Sciences, University of Alaska-Anchorage, Anchorage, AK, United States of America; 4 US Geological Survey Alaska Science Center, Anchorage, AK, United States of America; 5 UArctic, Ecology and Genetics Research Unit, University of Oulu, Oulu, Finland; Université de Sherbrooke, CANADA

## Abstract

With warmer springs, herbivores migrating to Arctic breeding grounds may experience phenological mismatches between their energy demands and the availability of high quality forage. Yet, how the timing of the start of the season and herbivore arrival influences forage quality is often unknown. In coastal western Alaska, approximately one million migratory geese arrive each spring to breed, where foliar %N and C:N ratios are linked to gosling survival and population growth. We conducted a three-year experiment where we manipulated the start of the growing season using warming chambers and grazing times using captive Pacific black brant (*Branta bernicla nigricans*) to examine how the timing of these events influences the quality of an important forage species. Our results suggest that grazing timing plays a much greater role than an advanced growing season in determining forage quality. All top models included grazing timing, and suggested that compared to typical grazing timing, early grazing significantly reduced foliar %C by 6% and C:N ratios by 16%, while late goose grazing significantly reduced foliar %N by 15% and increased foliar C:N ratios by 21%. While second-ranking top models included the effect of season, the advanced growing season effect was not significant and only reduced %N by 4%, increased %C by <1%, and increased C:N ratios by 5% compared to an ambient growing season. In summary, in years where geese arrive early, they will consume higher quality forage when they arrive and throughout the season, while in years that geese arrive late they will consume lower quality forage when they arrive and for the remainder of the season. When the growing season starts has only a minor influence on this pattern. Our findings suggest that cues determining migration and arrival times to breeding areas are important factors influencing forage quality for geese in western Alaska.

## Introduction

The phenology of species is changing, especially in the Arctic and northern regions where spring is beginning earlier and growing seasons are advancing [1, 2]. Advancement of the vegetative growing seasons can have consequences for migratory herbivores that have evolved to match the timing of migration and arrival to breeding areas with optimal resource availability [3, 4]. Some migratory species are tracking these changes, by both leaving wintering grounds earlier and arriving to breeding grounds earlier [5–7]. However, for other species, climate change is affecting the phenology of herbivores and their resources differently, resulting in what has been termed “phenological mismatch” [5, 8, 9]. Phenological mismatch is particularly likely for high-latitude, long-distance migrants because high latitudes are warming faster than lower latitudes, and long-distance migrants are using environmental cues that may not reflect the rapid changes at their destinations. Thus, it is expected that these species may arrive functionally “late” to northern breeding grounds [10–13].

Migratory geese are among the species most susceptible to phenological mismatch [14]. First, many goose species are long-distance migrants, often traveling from temperate, winter ranges (i.e., the Baja Peninsula and the Central Valley of California) to high latitude, summer breeding grounds (i.e., western Alaska) [7, 11, 15]. Second, geese, in particular, may be susceptible to mismatch because they are often unable to hasten their migration if they recognize they have migrated late, in part because they need to replenish resources en route [16, 17], and because many of their life phases at the breeding grounds, such as time between nest initiation and hatch, are relatively “fixed” [18, 19]. Finally, many migratory geese are mixed capital and income breeders, and thus depend on high quality, local resources at the breeding site for egg production upon arrival [20–22]. Therefore, determining whether earlier growing seasons and changes in the timing of arrival by geese results in lower quality forage for consumption is critical.

Phenologically late arrival by herbivores at breeding and rearing areas may result in lower quality forage consumption at a critical life history period and has been a focus of trophic interaction studies in changing climates [3]. These developing mismatches can have cascading consequences through herbivore physiology, fecundity, and juvenile mortality, and may be one of the primary drivers of population decline [3, 7, 12, 23–25]. While many studies propose that herbivores experiencing mismatch decline in abundance because late arriving herbivores only have access to low quality forage, only a handful of studies actually quantify forage quality (e.g., [10, 24, 26]). Rather, forage nutrition is often inferred from the timing of green-up, assuming that vegetation quality [i.e., leaf nitrogen (N) in particular] declines as the growing season progresses (e.g., [2, 7, 27, 28, 29]). However, the assumption that earlier growing seasons result in lower quality forage or a different seasonal pattern of forage N content needs to be tested [26].

It is expected that Arctic geese typically time their migration to match high leaf protein concentration in forage with post-hatch gosling growth [4, 30–32] because protein is the most limiting compound for gosling growth and new feather production. Protein is generally measured by N concentration in vegetation [4, 33, 34], but sometimes using carbon (C):N ratios [35–37]. Foliar N concentration is a more important indicator of forage quality than N biomass per m^2^ because geese have small gut volumes, rapid food passage, little ability to digest cellulose, and low efficiency at retaining N [38–41]. Thus, consuming more, lower quality vegetation cannot compensate for low N concentrations [42]. Experiments show that with a one-week later hatch date, goslings grow slower due to reduced foliar N concentrations [39, 41, 43], and that reduced gosling growth rates result in smaller body size at fledging, which has strong negative effects on subsequent survival and population recruitment [32, 33, 44–49]. For these reasons, changes in foliar N concentration is a valuable measure of breeding ground suitability and a key parameter in examining the potential effects of phenological mismatch on migratory geese.

The study of potentially developing phenological mismatches between geese and forage is complicated by the fact that goose grazing itself influences forage quality [50]. Goose herbivory increases forage quality by maintaining a shorter growth form (higher leaf N content), defecating unassimilated nutrients back into the soil, and trampling, which increases the rate of organic material breakdown [36, 50, 51]. While the presence and intensity of grazing has been well studied in Arctic goose grazing systems [50, 52–54], only two manipulative studies have examined how the timing of grazing may influence forage quality, and they have contradictory results. Person et al. [37], who similar to this study, investigated Pacific black brant (*Branta bernicla nigricans*) on the Yukon-Kuskokwim Delta, Alaska, found that plots that received early season grazing had higher foliar N concentrations compared to plots that only received late season grazing. This study suggests that early arrival to breeding grounds, and therefore early hatch and grazing, ensures that goslings have high quality forage. However, Beaulieu et al. [55] found in a system where the geese generally graze on low quality forage that neither early nor late season grazing by greater snow geese in Arctic Canada had an effect on foliar N concentrations. Thus, there may be a complicated relationship between the start of the growing season and the timing of goose grazing on forage quality.

While forage quality is often implicated in population declines in species experiencing phenological mismatch (e.g., [2, 7, 17, 27, 28, 56]), changes in forage quality as a result of an earlier growing season and changing times by migratory species has not been investigated in a controlled experimental setting. In this study, we took a novel approach to determine how changes in the timing of the growing season and timing of goose arrival influence the quality of an important goose forage species, *Carex subspathacea*, in western Alaska. More specifically, we conducted a manipulative full factorial experiment, where we changed the start of the growing season using warming chambers and the timing of goose herbivory using captive Pacific black brant, to determine how these factors influence forage quality (i.e., leaf %N, %C and C:N ratios). We hypothesized that earlier springs (i.e., an advanced growing season) would shift the peak in foliar percent N (peak of high quality forage) to earlier in the season, and potentially reduce season-long foliage quality [57, 58]. We expected that geese arriving earlier would experience higher quality forage than geese arriving late because the quality would be higher when they arrive and because quality may increase, or at least be maintained throughout the season, once geese begin grazing [37]. We discuss the implications of our results for goose populations.

## Materials and methods

### Study site

We conducted our experiment in a brackish, wet sedge meadow within 1 km of the coast on the active floodplain of the Tutakoke River in the Yukon-Kuskokwim (Y-K) Delta, western Alaska (61°15’N, 165°37’W; elevation 2 m). The Y-K Delta is 75,000 km^2^ of coastal wetlands and tundra along the Bering Sea. The landscape consists of emergent and submerged surficial deposits creating tidal mudflats in low-lying areas with brackish wet-sedge meadows at higher topographic positions. Snow and ice cover the region from late autumn to mid-spring, with no permafrost near the coast. The Bering Sea moderates the climate, with mean monthly temperatures ranging from -14°C to 10°C [59]. The years of our experiment were warmer than average, with mean monthly temperatures from May to July on average 0.5, 2.4, and 3.0°C higher for 2014, 2015, and 2016, respectively, than 90-year means (Bethel Station; 200 km from the site [60]).

The Y-K Delta is critical summer habitat for millions of migratory birds [61]. We chose to focus our grazing treatments on Pacific black brant for several reasons. First, they are common at the site and approximately half of all Pacific black brant nest on the Y-K Delta [62], with specific attention to the Tutakoke River colony, which has mean nest density of ca. 500 nests km^2^ [63]. Second, we expect that brant are the most susceptible geese at the site to an advanced growing season because they migrate further than other geese at the site; 50% winter in Baja California [64]. They are also smaller than other geese at the site, which means they probably have lower digestive efficiencies, and therefore are more impacted by changes in forage quality [65–67]. The ecology of cackling geese (*B*. *canadensis minima*) and emperor geese (*Anser canagicus*), during the summer months, is similar to brant in that they consume the same types of vegetation during brood rearing, hatch at similar times, and nest in similar regions [68, 69].

Brant are selective grazers and highly dependent on the sedge, *C*. *subspathacea*, during nesting and brood-rearing periods. This sedge grows in monoculture and is often referred to as ‘grazing lawn’ because goose grazing reduces it to a short stature that has higher foliar N concentration than the surrounding sedge, due largely to the grazing lawn being in a continual juvenile growth phase with no standing litter [36, 50]. While there are a few other plant species in this ecosystem with nutrient contents similar to grazing lawn, these other plants are dicots that occur singly or sparsely and therefore do not have enough landscape biomass to fuel the dietary needs of geese. Adult geese consume *C*. *subspathacea* as soon as it emerges; however, females do not feed substantially until midway through the typically 28-day incubation period (11 days between arrival and egg laying, 5–6 days for egg laying and 12 more days from the start to mid-incubation)[18]. The intensity of grazing increases following hatch when goslings begin to consume vegetation [70]. When goslings gain flight and adults regrow flight feathers, 40 days post-hatch for cackling geese, geese move inland to brood-rearing areas with concomitant declines in the use of grazing lawns [19]. Consequently, the period of maximum *C*. *subspathacea* grazing closely coincides with timing of hatch. Annual mean hatch date is highly correlated with nest initiation (R^2^ = 0.98) and arrival date [63, 71], and relatively inflexible because incubation length is largely fixed, and female brant initiate rapid ovarian follicular growth during the final leg of migration or immediately upon arrival at the nesting area [72].

**Fig 1:**
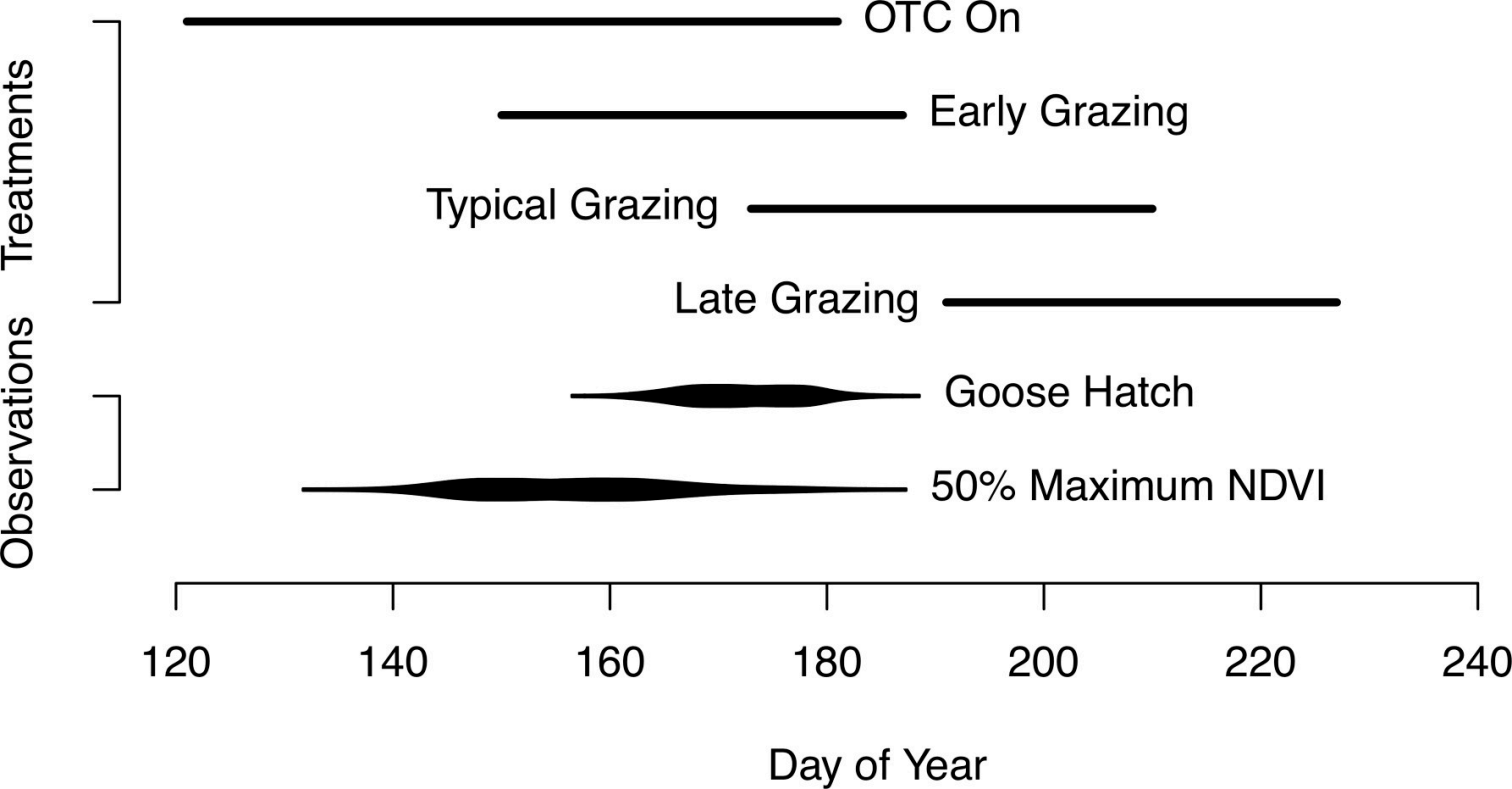
Timing of experimental treatments and observed events near the Tutakoke River in the Y-K Delta. Timing of experiment treatments for the advanced growing season [when the open-top chambers (OTCs) are on], and timing of late, typical and early grazing treatments. Hatch phenology data are from over 30 years (1984–2016) of direct observations [63, 77]. Also shown is the 50% maximum NDVI (normalized difference vegetation index) data from over 30 years (1982–2016) of observation [28]. Intensity of grazing increases following hatch when goslings begin to consume vegetation and females recover from nutrient deficits following nest incubation. Thus, early grazing treatments began shortly before the earliest mean hatch date. Typical grazing treatment started around the median hatch date. The late grazing treatment was timed to coincide with the latest mean hatch date.

Over the past 30 years, mean hatch has varied between 11-June and 30-June; median hatch date was 21-June, and the earliest and latest observed hatch was on 3-June and 9-July, respectively [63, 73](Fig 1). While the timing of hatch has varied over the past 30 years, the timing of green-up has similarly varied over the past 30 years. Using the day of year when the 50% maximum normalized difference vegetation index is achieved as a vegetation phenology metric, green-up varied between 23-May and 25-June in the Y-K Delta (D. Douglas unpublished data; methods follow [28])(Fig 1). While the relationship between hatch date and green-up is correlated (R^2^ = 0.78), for every day of season advancement, hatch date advances less than 0.5 days, leading to an expected greater mismatch in the future [74]. Recent observation of black brant near our site suggest they are declining by 2–4% per year [63, 75, 76].

### Experimental design

**Fig 2:**
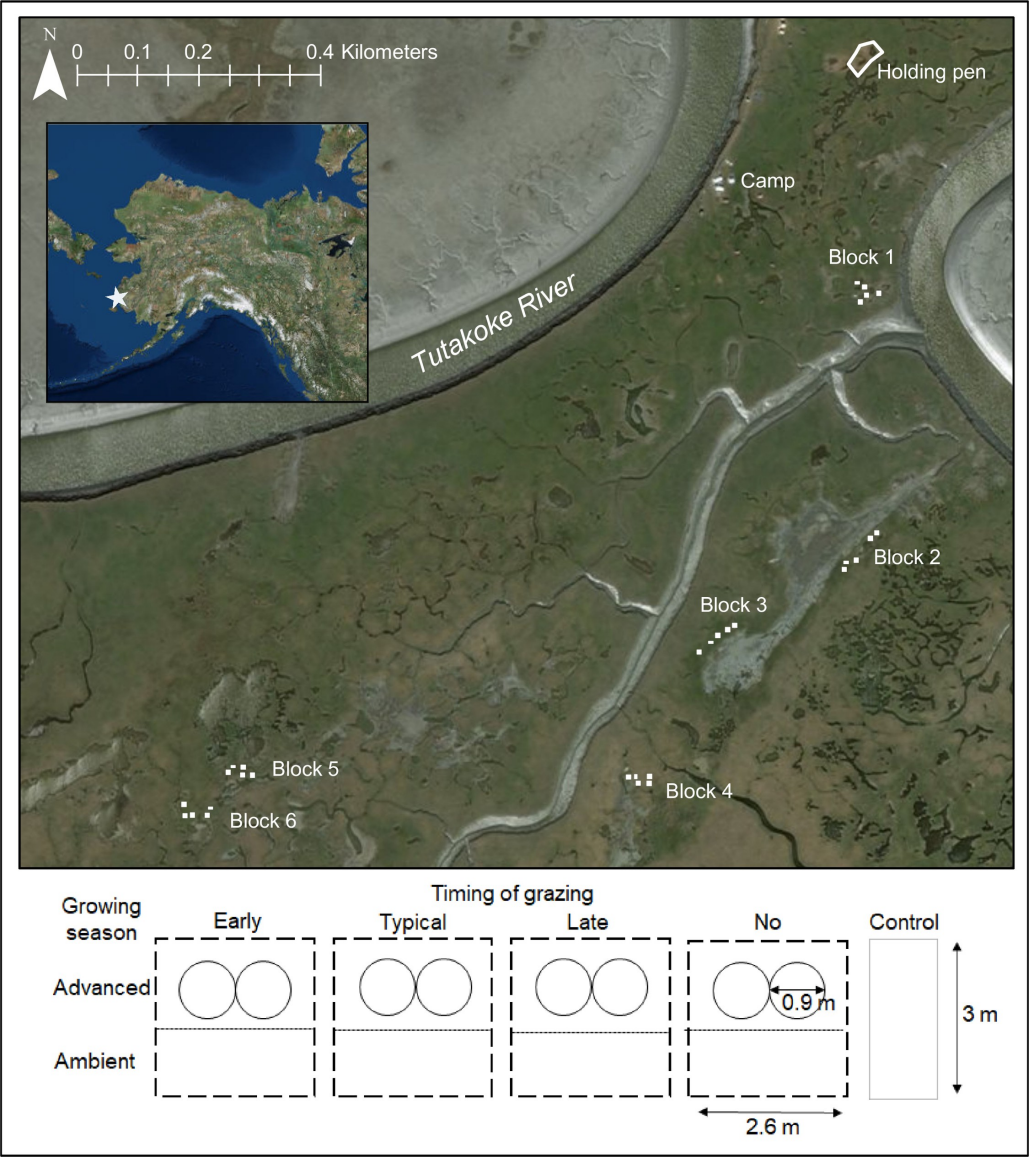
Experimental design. Image of the study site showing where the six blocks with the nine experimental treatments and the holding pen for geese were located. The bottom panel illustrates how the timing of grazing plots were placed together and fenced for the controlled grazing treatments. Created with ESRI ArcGIS 10.6.1 software. Basemap data sources include: Esri, DigitalGlobe, GeoEye, i-cubed, USDA, USGS, AEX, Getmapping, Aerogrid, IGN, IGP, swisstopo, and the GIS User Community.

We conducted a three-year experiment using a fully factorial design with two timing of growing season treatments (advanced and ambient) crossed with four timings of grazing treatments (early, typical, late, and no grazing) for a total of eight treatments, plus a background grazing control, where wild geese were allowed to graze naturally (Fig 2). Our factorial crossings of growing seasons and goose grazing times simulated scenarios of phenological mismatch, where the growing season was advanced by three weeks (see below) or not, and goose grazing was advanced by three weeks, not changed, or delayed by three weeks (Table 1). The ‘no grazing’ treatment represents a scenario where goose populations fail to arrive at the breeding ground.

**Table 1:** Experiment treatments. Season indicates timing of season advancement treatment: 3 weeks early (-3) and was ambient (0). Grazing indicates timing of goose grazing treatment: 3 weeks early (-3), typical (0) and 3 weeks late (+3). A negative mismatch indicates an advanced season relative to grazing; positive mismatch indicates advanced grazing relative to the season. There are more negative mismatches than positive because we only advanced the growing season while we advanced and delayed grazing. N/A = no mismatch because no goose arrival occurred.

Treatment	Season	Grazing	Season	Grazing	Mismatch	Explanation
1	Advanced	Early	-3	-3	0	Season is 3 weeks early and grazing is 3 weeks early, resulting in no mismatch
2	Ambient	Early	0	-3	+3	Season unchanged and grazing 3 weeks early, resulting in 3 weeks of mismatch
3	Advanced	Typical	-3	0	-3	Season is 3 weeks early and grazing is typical time, resulting in 3 weeks of mismatch
4	Ambient	Typical	0	0	0	Season unchanged and grazing is typical time, resulting in no mismatch (represents current system)
5	Advanced	Late	-3	+3	-6	Season is 3 weeks early and grazing is 3 weeks late, resulting in 6 weeks of mismatch
6	Ambient	Late	0	+3	-3	Season unchanged and grazing is 3 weeks late, resulting in 3 weeks of mismatch
7	Advanced	None	-3	N/A	N/A	Season is 3 weeks early but geese do not arrive, resulting in no mismatch
8	Ambient	None	0	N/A	N/A	Season unchanged but geese do not arrive, resulting in no mismatch

We had six replicate blocks with each treatment for a total of 54 plots. Measurements were made inside plots that were 1.7 m x 0.85 m. We established plots in April 2014 and we applied treatments from 1-May through 15-August for three years. We installed fencing around all experimental plots, except the background grazing control plots, to exclude wild goose grazing.

To advance the growing season, we used two conical passive-warming open-top chambers (OTCs; 30 cm height x 85 cm base dia. x 50 cm top dia.) placed adjacent to each other [78]. We placed OTCs on plots 1-May and left them on to advance the growing season, until 1-July, each year (Fig 1). We temporarily removed chambers before 1-July during grazing treatments (see below), as appropriate. We monitored air and soil temperature (10-cm above- and belowground) in every plot using iButton microloggers (models DS1921G/Z, Maxim Integrated, San Jose, CA). From 1-June to 1-July, OTCs warmed plots 10-cm aboveground, on average, between 0.6 and 1.7°C and 10-cm belowground, on average, between 0.6 and 1.0°C. Following OTC removal, temperature differences were < 0.3°C between advanced and ambient treatments. While OTCs are often used to increase temperature, OTCs also accelerate growth at the start of the season [79, 80]. We monitored season advancement by measuring the height of green vegetation on 10 shoots in a fixed 10 cm x 10 cm quadrant in each plot every 2–3 weeks in 2014, and weekly in 2015 and 2016.

To manipulate the timing of grazing, we constructed fenced exclosures (2.6 m x 3.0 m) around paired advanced and ambient growing season plots, and introduced wild-caught geese into the exclosures at particular times during the season (Fig 2). We initiated the early, typical and late grazing treatments on 30-May, 20-June, and 9-July, respectively, to approximate the 30-day variation in the range of hatch dates (3-June to 9-July) observed over the past three decades in the Tutakoke River colony [63, 73](Fig 1). Further, we selected these dates to account for the logistical challenges of using actual, as opposed to simulated, goose grazing in our experiments (treatments had to start after we could collect geese on nests in late May), and to avoid overlapping all three grazing timing treatments, which would have required considerably more geese. Prior to the start of grazing treatments each year, we captured ca. 20 female Pacific black brant for creating these treatments and held them for the summer in a fenced area after clipping flight feathers.

Importantly, each grazing treatment only differed in when the treatment was initiated; total available grazing time was the same for each treatment. Grazing treatments (early, typical, and late grazing) consisted of two geese that we allowed to graze, trample and defecate for 24-h, four times, each separated by 12 days to simulate the ca. 40 days of intense post-hatch grazing [19]. We created the same grazing intensity in each treatment, 7.2 goose-hours m^-2^ month^-1^, to be similar to a previous controlled-grazing study in the same population of geese [81]. Prior to each treatment, we held geese without food for two hours to allow feces from captive feed to pass [82]. After completion of the 24-h treatments, we held birds for an additional two hours and we returned deposited feces to appropriate experimental plots. When not used in grazing treatments, we allowed geese to graze freely on natural vegetation in a fenced area, supplemented with goose feed. We released geese at the end of the experiment after they regained flight feathers.

Throughout the three-year experiment, we destructively harvested aboveground biomass every three weeks during the growing season from a randomly selected, but unique, 5 cm x 5 cm area in each plot. We separated aboveground live and dead vegetation, washed the vegetation free of soil, dried it at 60°C to constant weight, and weighed it. We then ground and homogenized live vegetation samples with a 20-mesh size Wiley Mill and analyzed the samples for %N and %C (N and C concentrations) using a PDZ Europa ANCA-GSL elemental analyzer interfaced to a PDZ Europa 20–20 isotope ratio mass spectrometer (Sercon Ltd., Cheshire, UK) at the UC Davis Stable Isotope Facility.

### Statistical analyses

First, we examined the effectiveness of OTCs to advance the growing season using vegetation height data as the response variable. We used mean stem height data because we had the most data for this variable before 1-July. The model included categorical predictors of year and treatment (ambient or advanced), a continuous predictor of day of year (DOY), all interactions, and a random plot within block effect. We restricted data to plots that did not experience grazing before 1-July to remove the effect of grazing. We used regression coefficients to calculate the number of days needed for ambient plots to reach the same height as advanced plots. Leffler et al. [74] present this analysis in more detail, but we present the results here for completeness.

Next, we examined the effectiveness of our grazing treatments to simulate background-grazing levels using aboveground biomass as the response variable. We used aboveground biomass for this analysis because we felt it was a better response variable than height to describe the effects of grazing, and we had sufficient data because this is a season-long analysis as opposed to just prior to OTC removal. We limited the included data to plots with the ambient growing season treatment and the background grazing control. The model included the categorical goose grazing treatments and a continuous predictor of DOY, their interaction, and a random plot with block effect. We ran each year separately. This analysis is presented in more detail in Choi et al. [83], but we present it here for completeness.

Then, we tested the effects of timing of the treatments, growing season (advanced, ambient) and timing of goose grazing (early, typical, late, no grazing), on forage quality. We used forage quality variables (foliar %N, foliar %C, and foliar C:N) as continuous response variables, experimental treatments (growing season timing and goose grazing timing) and year as as categorical and DOY as a continuous fixed effect predictor variable, and treated plot within block as a random effect. We limited model combinations to include interactions with no more than two predictor variables because we did not think the three-way interaction would be biologically meaningful, and determined the most important variables by presence in the top-performing model. We included a first-order autocorrelation structure to account for repeated measures within subjects over time. We arcsine-square root transformed %N and %C data, and log-transformed C:N ratios prior to analysis to meet assumptions of normality and homogeneity of variance.

For these analyses, we employed a linear mixed model framework with model selection using Akaike Information Criteria (AIC). We fit models using the nlme package within the R statistical computing environment (Pinheiro et al. 2017, R Core Development Team). We determined top models using ΔAIC and considered models to be similar if ΔAIC< 2 [84]. Using the summary function in nlme package, we determined parameter estimates of the fixed effects. We focus our discussion on variables that are significantly different from the reference intercept (ambient season, typical grazing) in the top models (S1 and S2 Tables). Percent changes that we give in this manuscript represent significant differences in means across the third year of the experiment, but we observed similar results every year of the experiment (see S3 Table).

## Results

### Treatment effectiveness

In our experiment, OTCs advanced the growing season each year by ca. 20 days, measured as taller vegetation heights and greater growth rates. More specifically, modeled rates of growth indicate the season was advanced by 22, 18, and 21 days at the end of June 2014, 2015, and 2016, respectively (for more details see [74]).

Aboveground biomass in the typical goose grazing plots in years 1 and 2, and the early grazing plots in all three years, were not different than background control plots (for more details see [83]). We based treatments on historic 30-year mean grazing times, but the three years of our experiment were three of the earliest six hatch dates on record for the Y-K Delta, while the third year had the earliest mean hatch date on record (11-June) [63]. Given these conditions, we expected background control plots, which experienced wild goose grazing, to have aboveground biomass more similar to early grazing plots than the typical grazing plots by year 3.

### Treatment analysis

For foliar %N, %C, and C:N ratios, the top model included year and an interaction between grazing treatments and day of year (DOY) (Table 2). For foliar %N, %C, and C:N ratios, the second ranked top-performing model, with a ΔAIC < 2, included these variables plus the effect of season. Investigation of the fixed effects for the top-performing and second-ranked models showed the same factors and interactions as significantly different from the reference treatment, and season was never significant in the second-ranking model (see S1 and S2 Tables).

**Table 2:** Four top-performing models for foliage chemistry. Top models are based on AIC model selection for the treatment analysis over three years (2014–16). Abbreviations: Grazing = Timing of grazing treatment, Season = Timing of season treatment, DOY = Day of year, Year = Year of experiment, AIC = Akaike information criterion, logLik = log likelihood, df = degrees of freedom.

Model	logLik	AIC	ΔLogLik	ΔAIC	df	Weight
**Foliar %N**						
Year + Grazing*DOY	1774.6	-3521.2	150.3	0.0	14	0.530
Year + Grazing*DOY **+** Season	1775.3	-3520.5	151.0	0.7	15	0.420
Year*Grazing + DOY	1756.7	-3479.3	132.4	41.8	17	<0.001
Year*Grazing + Season + DOY	1757.4	-3478.8	133.1	42.4	18	<0.001
**Foliar %C**						
Year + Grazing*DOY	1278.7	-2529.5	72.4	0.0	14	0.700
Year + Grazing*DOY + Season	1278.9	-2527.7	72.5	1.7	15	0.300
Grazing*DOY	1260.9	-2497.8	54.6	31.7	12	<0.001
Grazing*DOY + Season	1261.0	-2496.0	54.7	33.4	13	<0.001
**Foliar C:N**						
Year + Grazing*DOY	222.1	-416.2	164.7	0.0	14	0.560
Year + Grazing*DOY + Season	222.9	-415.7	165.5	0.5	15	0.440
Grazing*DOY	195.0	-365.9	137.6	50.3	12	<0.001
Grazing*DOY + Season	195.7	-365.4	138.3	50.9	13	<0.001

**Fig 3:**
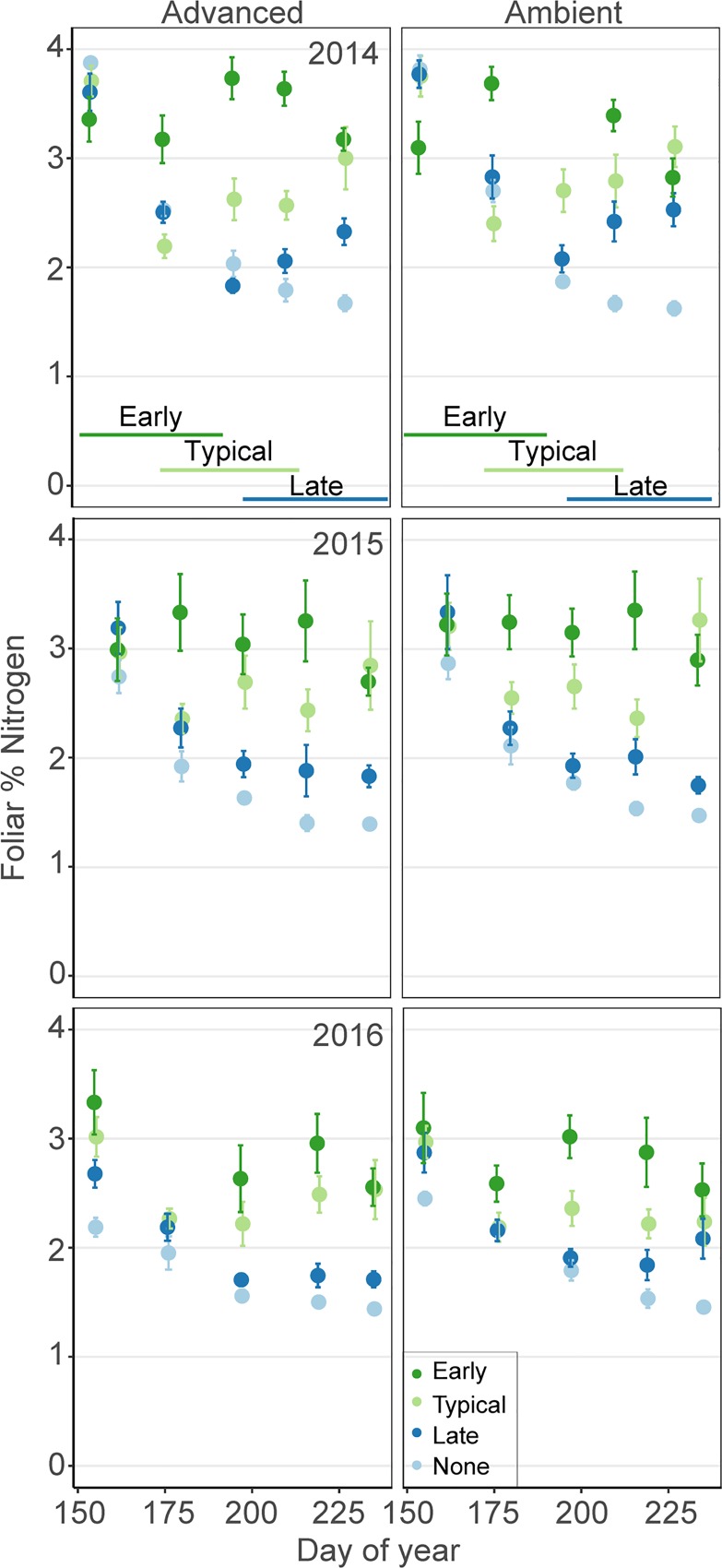
Foliar percent N. Mean percent N (± 1 SE) for treatment plots across the growing season from 2014–2016 (n = 6 replicates/treatment). Treatments included advanced and ambient growing seasons, and early, typical, late and no grazing. Lines on the bottom show the timing of the early, typical and late grazing treatments.

Foliar %N generally decreased in each year of the experiment in all treatments. The only grazing treatment that did not significantly interact with DOY was early grazing (S1 Table), because %N remained high over the season in this treatment. Averaged across year three, early grazing maintained foliar %N at a level similar to that in the typical grazing, while late and no goose grazing led to lower foliar %N values by 15% and 26%, respectively (Fig 3). While season is present in the second-ranking model, the advanced growing season led to only a small decrease in %N by 4% (S3 Table) and the fixed effect of season was not significant (S2 Table).

**Fig 4:**
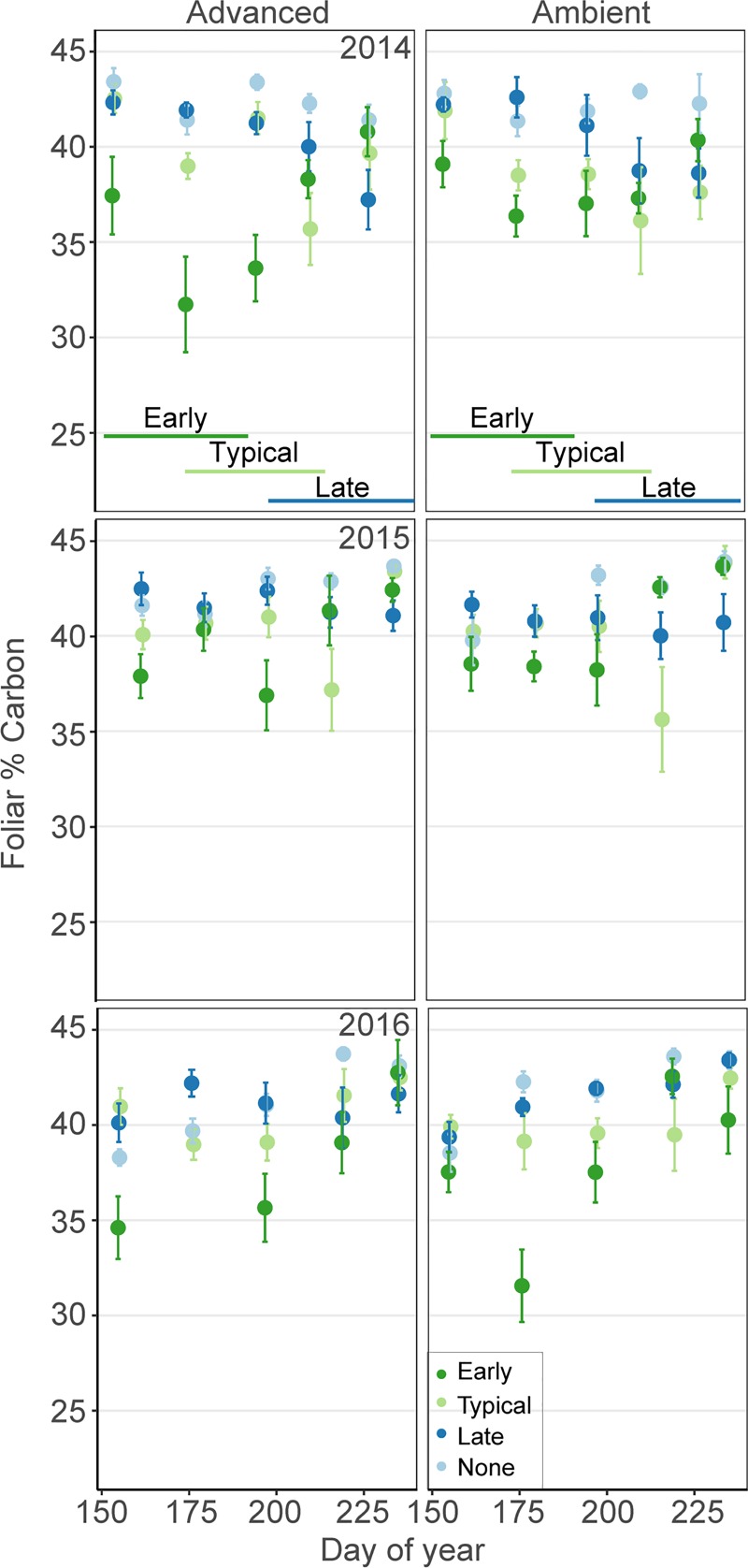
Foliar percent C. Mean percent C (± 1 SE) for treatment plots across the growing season from 2014–2016 (n = 6 replicates/treatment). Treatments included advanced and ambient growing seasons, and early, typical, late and no grazing. Lines on the bottom show the timing of the early, typical and late grazing treatments.

Foliar %C was highest in the second year. The late grazing treatment did not interact with DOY (S2 Table), because %C remained relatively high over the season in this treatment. Averaged across year three, early grazing reduced foliar %C by 6% compared to typical grazing, while no goose grazing increased foliar %C by 3% (Fig 4). While season showed up in the second-ranking model, the advanced growing season led to only a small increase in %C by <1% (S3 Table) and the fixed effect of season was not significant (S2 Table).

**Fig 5:**
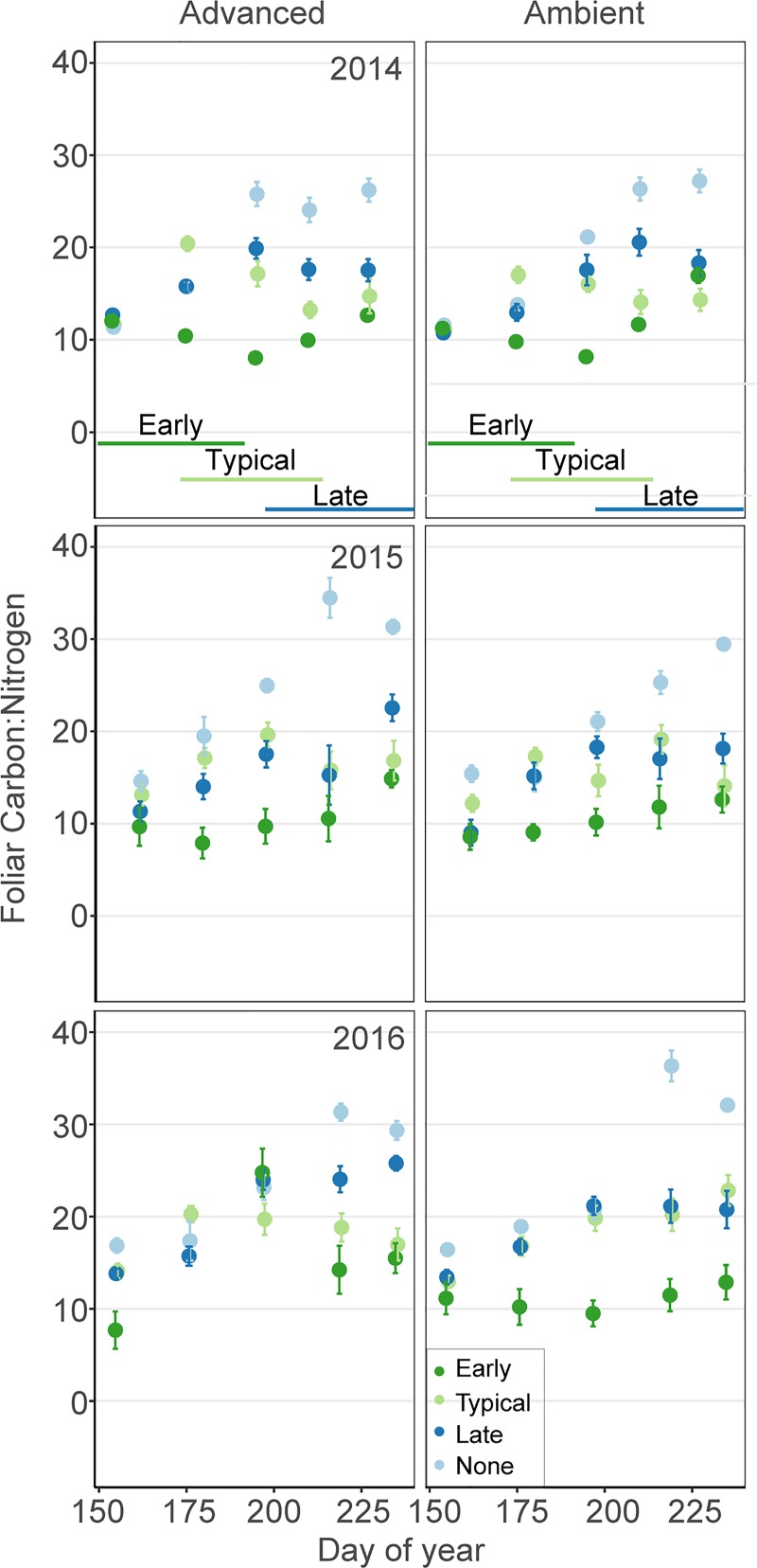
Foliar C:N ratios. Mean C:N ratios (± 1 SE) for treatment plots across the growing season from 2014–2016 (n = 6 replicates/treatment). Treatments included advanced and ambient growing seasons, and early, typical, late and no grazing. Lines on the bottom show the timing of the early, typical and late grazing treatments.

Foliar C:N values increased each year of the experiment. All grazing treatments significantly interacted with DOY (S2 Table). Averaged across year three, early goose grazing decreased foliar C:N ratios by 16%, while late and no goose grazing increased foliar C:N ratios by 21% and 41%, respectively, compared to typical grazing (Fig 5). Again, season was in the second-ranking model, but the advanced growing season led to only a relatively small increase in C:N ratios by ca. 5% (S3 Table) and the fixed effect of season was not significant (S2 Table).

## Discussion

We designed our experiment to investigate how changes in the timing of the growing season and changes in the timing of grazing influence forage quality of *C*. *subspathacea*, an important forage species for geese on the Y-K Delta. We found that the initiation of grazing had a much greater effect on forage quality than earlier springs, even though the shifts in the start of growing season and the timing of grazing were equivalent (ca. 3 weeks). We found that grazing 3-weeks earlier (i.e., early arrival) increases season-long forage quality by decreasing C:N ratios (by 16%) and a 3-week delay in grazing (i.e., late arrival) decreases season-long forage quality by decreasing N concentrations (by 15%). Simultaneously, our results indicate that earlier springs decrease forage quality, but they have a small, and insignificant, influence on forage quality compared to timing of grazing. In conclusion, our results suggest that we cannot assume herbivores experience lower quality forage simply based on the timing of green-up relative to herbivore arrival time.

In our system, goose herbivory influences the structure and quality of the vegetation [36, 50]. Therefore, it was not surprising that timing of grazing played such an important role in influencing season-long forage quality. We found that early grazing increased season-long forage quality by decreasing foliar %C by 6% and C:N ratios by 17%, but did not increase season-long %N significantly compared to typical grazing. Alternatively, late grazing decreased season-long forage quality as indicated by a 15% decline in foliar %N and a 22% increase in C:N. Our results are similar to the findings of Person et al. [37] who found standing crop N increased and C:N ratios decreased with early grazing compared to late grazing. According to our results, if it is %N that matters most for geese, it is late arrival that has the greatest potential negative effect on geese. Considering that gosling size, and presumably recruitment, is significantly influenced by the drop in foliar %N with a one-week delay in grazing [39, 41, 43], our observed reduction in foliar %N by 15% with a three-week grazing delay would likely have large negative repercussions on gosling growth, the most important predictor of juvenile survival and a critical contributor to population growth [76, 85].

The influence of timing of grazing was not restricted to season-long impacts on foliage quality. Nearly all of our fixed effects had significant interactions between grazing treatments and day of year (DOY; Table 2), usually reflecting the fact that shortly after each of the grazing treatments would start, there would be both an increase in %N and a decrease in %C. Thus, geese need to initiate grazing to prevent the steady decline in forage quality observed in the no-grazing treatment. However, top models did not have significant interactions between grazing treatment and DOY for: early grazing on %N and late grazing on %C (S1 Table). This is because with early grazing, %N remains high throughout the season (Fig 3). This is important because it indicates there will be higher quality forage when gosling hatch and start feeding themselves, potentially the life stage most dependent on %N. Further, geese that arrive late only experience comparatively low quality forage (high %C), as it is low when they arrive and it does not increase to typical-grazing quality after geese initiate grazing (Fig 4 and S1 Table). Further, by year 3, the difference in foliar %N first experienced by gosling hatching late is 40% lower than those hatching early (1.8%N vs. 3.0%N).

**Fig 6:**
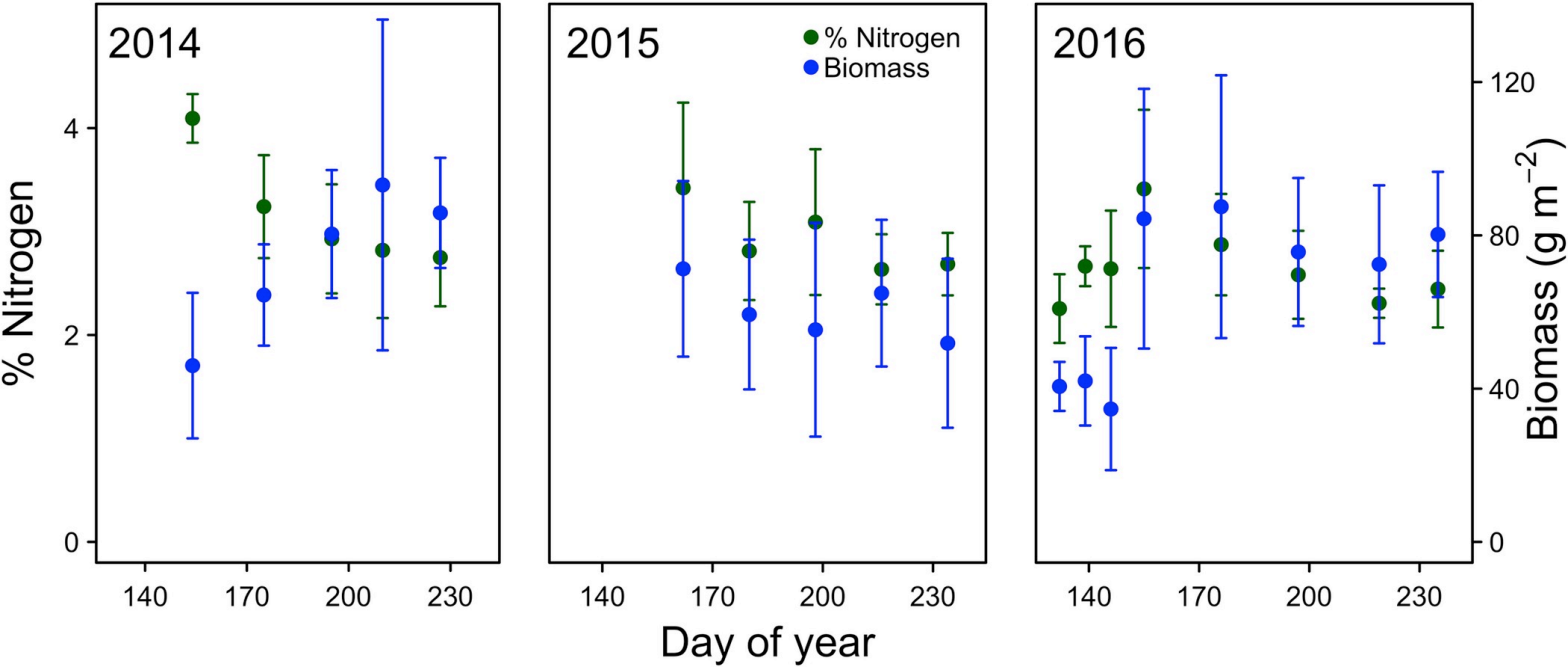
Foliar %N and aboveground biomass for control plots. Means (± 1 SE) from 2014–2016 are presented (n = 6 replicates/treatment) for our naturally grazed control plots over the growing season.

For all treatments (except early grazing), the peak in forage quality, as measured by maximum %N, appears at the start of the season, ca. day 155 (4-June). Our results suggest that early grazing can shift the peak in quality, such that there is still an early peak, but also a subsequent peak around day 200 (19-July), thus ensuring season-long high quality forage (Fig 3). In the other treatments, the timing of the peak does not appear to be a function of the lack of data prior to this date, because in the background grazing control plots, we also observed a season peak ca. day 160, despite having data in year 3 starting day 130 (10-May) (Fig 6). Peak hatch during our three year experiment, mean day 165 (14-June), is only ca. 5 days after the peak in forage quality in our background control plots (Figs 1 and 5), providing support for the idea that geese time migration to match forage quality with post-hatch gosling growth [30–32, 70]. Our experimental dataset, which includes five data points per year for three years, represents one of the most complete datasets we know of measuring changes in forage quality with phenological change, but limits interpretation. Future studies with more frequent data collection could elucidate these changes even further.

The result that the timing of growing season plays a smaller role than timing of grazing on forage quality in this system is important. Most studies on phenological mismatch assume that if the growing season starts earlier, the quality of forage will be reduced by the time migratory species arrive, either because the decline in forage quality has accelerated or because the earlier growing season has moved the peak in high quality forage earlier [3]. It is well known that forage quality declines over the growing season [24, 29, 86]. Indeed, we found in our no-grazing treatment a continuous and cumulative decline in %N and an increase in %C as the season progressed. But it is less well-known how earlier growing seasons influence forage quality [26]. Our results suggest that a 3-week season advancement in a veritable sedge monoculture only creates a small, not significant, decrease in foliage quality, and does not shift the peak in forage quality to earlier in the season (no DOY and season interactions; Table 2). These results are surprising because we did find that plants grew taller and faster in our advanced season treatment, although the increase in height did not translate to higher season-long aboveground biomass [83] or greater C uptake [74, 87].

Many studies do not measure the quality of forage when herbivores arrive, but rather use the start of the growing season and arrival time of herbivores, and the assumption that there is seasonal decline in forage quality to determine if a mismatch is developing (e.g., [2, 7, 17, 27, 28, 29]). One study found that forage quality did not change with an advanced growing season, and hence concluded there was no evidence of mismatch for migratory caribou [26]. Like this study, our findings call into question the assumption that forage quality will necessarily be significantly lower if seasons advance, and whether this can be used to assume that a phenological mismatch is occurring. We thus caution for instance that NDVI metrics, that are used to discern “green-up” and possible changes in the seasonality of forage quality for herbivores, be validated with on-the-ground measures of plant nutrition [88, 89]. It should be noted that our results only reflect an advanced season from early season warming, while season-long warming may shift %N earlier or lower plant quality [90], as has been found in a similar goose grazing system, although primarily well after hatch [58].

Possible explanations for only small decreases in forage quality (i.e., leaf %N) over the growing season when spring is early are that: a) Arctic warming is leading to greater summer-long soil decomposition, N-mineralization, and availability of inorganic N [91]; or b) summer-long access to and use of organic N sources [92], that may be derived in part from fecal N, is being rapidly assimilated by plants. We have evidence that fecal N may be partially involved in maintaining leaf N and thus forage quality later into the summer, as the δ^15^N value of *C*. *subspathacea* is enriched (from ~1.5 to 3 per mil) in areas where geese are grazing and their feces are left to decompose as opposed to areas where feces are removed (unpubl. data). This also may in part explain why late season grazing has lower %N in leaves. These summer soil and plant mineral nutrition traits may also be dependent upon prior winter snow conditions [29], and greater winter N mineralization rates when snow is deep [93] providing a buffer of soil N to support longer growing season leaf N.

## Conclusions

In summary, we found that the ability of migratory geese to arrive early and initiate grazing in their breeding ground plays a larger role than earlier springs in regulating forage quality and maintaining the trophic cohesion between brant geese and plants in the “New Arctic”. While seasons are already advancing, and the three years of our experiment were warmer than average, our results suggest that a 3-week season advancement will not greatly reduce forage quality at this time. However, if geese are receiving and using erroneous signals regarding when to leave their winter habitat and they arrive at the breeding ground late in the growing season, this will influence the quality of forage on the landscape when they arrive. Further, their grazing will likely not be sufficient to stimulate enough new, high leaf N vegetation to improve forage quality in a late arrival scenario.

Despite the potential negative consequences of late arrival and grazing, there are multiple possible future scenarios for the Y-K Delta that would likely have less negative effects on geese. Though Pacific black brant are generally philopatric to breeding and wintering sites [94–96], they may be able to alter their behavior and use alternate breeding sites, or winter farther north [97, 98]; thus, it is not clear that they will arrive late in the future [17]. In fact, the 3 years of our study were three of the earliest 6 years for mean hatch date on record, and the final year of our experiment was the earliest mean hatch date out of the 30-year record [63], illustrating the potential for geese to adjust their arrival times earlier. Our results suggest that early goose arrival will help maintain high forage quality regardless of the timing of the growing season. If geese continue to arrival early, our results suggest that this is will not affect their populations negatively because early arriving geese will experience lower %C and C:N ratios in forage.

## Supporting information

S1 Table Fixed effects of the top-performing model. The reference level for the models (i.e., the intercept) was treatment: ambient growing season, typical grazing timing in 2014. Effects not listed did not show up in the top models. Abbreviations: SE = standard error, Early = early grazing, Late = late grazing, None = no grazing, DOY = day of year. Foliar %N and %C values were arcsine square-root transformed; foliar C:N values were log-transformed. Bolded values are significant.
(DOCX)

S2 Table Fixed effects of the second-ranked top-performing models. The reference level for the models (i.e., the intercept) was treatment: ambient growing season, typical grazing timing in 2014. Effects not listed did not show up in the top models. Abbreviations: SE = standard error; Early = early grazing, Late = late grazing, None = no grazing, DOY = day of year, Advanced = advanced growing season. Foliar %N and %C values were arcsine square-root transformed; foliar C:N values were log-transformed. Bolded values are significant.
(DOCX)

S3 Table Mean percent changes by treatment for each year. The reference level was the ambient growing season, typical grazing timing treatment. Abbreviations: Early = early grazing, Late = late grazing, None = no grazing, Advanced = advanced growing season treatment.
(DOCX)
