# Supplementary material for: Migratory goose arrival time plays a larger role in influencing forage quality than advancing springs in an Arctic coastal wetland
Source: PLoS One. 2019 Mar 13;14(3):e0213037. doi: 10.1371/journal.pone.0213037 (PMC6415786; doi:10.1371/journal.pone.0213037)
Supplement: S1 Table — The reference level for the models (i.e., the intercept) was treatment: ambient growing season, typical grazing timing in 2014. Effects not listed did not show up in the top models. Abbreviations: SE = standard error, Early = early grazing, Late = late grazing, None = no grazing, DOY = day of year. Foliar %N and %C values were arcsine square-root transformed; foliar C:N values were log-transformed. Bolded values are significant. (DOCX) [file pone.0213037.s001.docx]

**Supplemental Table 1. Fixed effects of the top-performing model.** The reference level for the models (i.e., the intercept) was treatment: ambient growing season, typical grazing timing in 2014. Effects not listed did not show up in the top models. Abbreviations: SE = standard error, Early = early grazing, Late = late grazing, None = no grazing, DOY = day of year. Foliar %N and %C values were arcsine square-root transformed; foliar C:N values were log-transformed. Bolded values are significant.

|  |  | **Foliar %N** | | |  | **Foliar %C** | | |  | **Foliar C:N** | | |
| --- | --- | --- | --- | --- | --- | --- | --- | --- | --- | --- | --- | --- |
| **Effect** |  | **Value** | **SE** | **P** |  | **Value** | **SE** | **P** |  | **Value** | **SE** | **P** |
| **Intercept** |  | **0.191** | **0.0010** | **<0.001** |  | **0.652** | **0.021** | **<0.001** |  | **2.27** | **0.129** | **<0.001** |
| **2015** |  | **-0.006** | **0.0014** | **<0.001** |  | **0.020** | **0.0032** | **<0.001** |  | **0.123** | **0.022** | **<0.001** |
| **2016** |  | **-0.012** | **0.0014** | **<0.001** |  | **0.014** | **0.0032** | **<0.001** |  | **0.184** | **0.022** | **<0.001** |
| **Early** |  | 0.010 | 0.013 | 0.416 |  | **-0.173** | **0.029** | **<0.001** |  | **-0.572** | **0.170** | **0.002** |
| **Late** |  | **0.048** | **0.013** | **0.001** |  | 0.032 | 0.028 | 0.272 |  | **-0.466** | **0.168** | **0.008** |
| **None** |  | **0.060** | **0.013** | **<0.001** |  | **-0.074** | **0.028** | **0.013** |  | **-0.972** | **0.168** | **<0.001** |
| **DOY** |  | **-1.08E-04** | **4.42E-05** | **0.015** |  | 8.31E-05 | 1.00E-04 | 0.407 |  | **0.00167** | **5.75E-04** | **0.004** |
| **Early*DOY** |  | 1.57E-05 | 6.25E-05 | 0.802 |  | **8.16E-04** | **1.42E-04** | **<0.001** |  | **0.00198** | **8.20E-04** | **0.016** |
| **Late*DOY** |  | **-3.13E-04** | **6.19E-05** | **<0.001** |  | -1.01E-04 | 1.40E-04 | 0.471 |  | **0.00344** | **8.09E-04** | **<0.001** |
| **None*DOY** |  | **-4.36E-04** | **6.19E-05** | **<0.001** |  | **4.88E-04** | **1.40E-04** | **0.001** |  | **0.00697** | **8.09E-04** | **<0.001** |
