# Supplementary material for: Migratory goose arrival time plays a larger role in influencing forage quality than advancing springs in an Arctic coastal wetland
Source: PLoS One. 2019 Mar 13;14(3):e0213037. doi: 10.1371/journal.pone.0213037 (PMC6415786; doi:10.1371/journal.pone.0213037)
Supplement: S2 Table — The reference level for the models (i.e., the intercept) was treatment: ambient growing season, typical grazing timing in 2014. Effects not listed did not show up in the top models. Abbreviations: SE = standard error; Early = early grazing, Late = late grazing, None = no grazing, DOY = day of year, Advanced = advanced growing season. Foliar %N and %C values were arcsine square-root transformed; foliar C:N values were log-transformed. Bolded values are significant. (DOCX) [file pone.0213037.s002.docx]

**Supplemental Table 2. Fixed effects of the second-ranked top-performing models.** The reference level for the models (i.e., the intercept) was treatment: ambient growing season, typical grazing timing in 2014. Effects not listed did not show up in the top models. Abbreviations: SE = standard error; Early = early grazing, Late = late grazing, None = no grazing, DOY = day of year, Advanced = advanced growing season. Foliar %N and %C values were arcsine square-root transformed; foliar C:N values were log-transformed. Bolded values are significant.

|  |  | **Foliar %N** | | |  | **Foliar %C** | | |  | **Foliar C:N** | | |
| --- | --- | --- | --- | --- | --- | --- | --- | --- | --- | --- | --- | --- |
| **Effect** |  | **Value** | **SE** | **P** |  | **Value** | **SE** | **P** |  | **Value** | **SE** | **P** |
| **Intercept** |  | **0.192** | **0.0098** | **<0.001** |  | **0.651** | **0.021** | **<0.001** |  | **2.26** | **0.130** | **<0.001** |
| **2015** |  | **-0.006** | **0.0014** | **<0.001** |  | **0.020** | **0.0032** | **<0.001** |  | **0.123** | **0.0221** | **<0.001** |
| **2016** |  | **-0.012** | **0.0014** | **<0.001** |  | **0.014** | **0.0032** | **<0.001** |  | **0.184** | **0.0224** | **<0.001** |
| **Early** |  | 0.011 | 0.013 | 0.415 |  | **-0.17** | **0.029** | **<0.001** |  | **-0.572** | **0.170** | **0.002** |
| **Late** |  | **0.048** | **0.013** | **<0.001** |  | 0.032 | 0.028 | 0.273 |  | **-0.466** | **0.168** | **0.008** |
| **None** |  | **0.060** | **0.013** | **<0.001** |  | **-0.074** | **0.028** | **0.013** |  | **-0.972** | **0.168** | **<0.001** |
| **DOY** |  | **-1.08E-04** | **4.42E-05** | **0.015** |  | 8.30E-05 | 1.00E-04 | 0.407 |  | **0.0017** | **5.76E-04** | **0.004** |
| **Advanced** |  | -0.002 | 0.002 | 0.253 |  | 0.002 | 0.003 | 0.615 |  | 0.037 | 0.030 | 0.228 |
| **Early*DOY** |  | 1.56E-05 | 6.26E-05 | 0.804 |  | **8.16E-04** | **1.42E-04** | **<0.001** |  | **0.0020** | **8.21E-04** | **0.016** |
| **Late*DOY** |  | **-3.13E-04** | **6.19E-05** | **<0.001** |  | -1.01E-04 | 1.41E-04 | 0.471 |  | **0.0034** | **8.10E-04** | **<0.001** |
| **None*DOY** |  | **-4.36E-04** | **6.19E-05** | **<0.001** |  | **4.88E-04** | **1.41E-04** | **0.001** |  | **0.0070** | **8.10E-04** | **<0.001** |
