# Supplementary material for: Migratory goose arrival time plays a larger role in influencing forage quality than advancing springs in an Arctic coastal wetland
Source: PLoS One. 2019 Mar 13;14(3):e0213037. doi: 10.1371/journal.pone.0213037 (PMC6415786; doi:10.1371/journal.pone.0213037)
Supplement: S3 Table — The reference level was the ambient growing season, typical grazing timing treatment. Abbreviations: Early = early grazing, Late = late grazing, None = no grazing, Advanced = advanced growing season treatment. (DOCX) [file pone.0213037.s003.docx]

**Supplemental Table 3. Mean percent changes by treatment for each year.** The reference level was the ambient growing season, typical grazing timing treatment. Abbreviations: Early = early grazing, Late = late grazing, None = no grazing, Advanced = advanced growing season treatment.

|  |  | **Foliar %N** | | |  | **Foliar %C** | | |  | **Foliar C:N** | | |
| --- | --- | --- | --- | --- | --- | --- | --- | --- | --- | --- | --- | --- |
| **Effect** |  | **2014** | **2015** | **2016** |  | **2014** | **2015** | **2016** |  | **2014** | **2015** | **2016** |
| **Early** |  | 20% | 14% | 14% |  | -5% | -1% | -6% |  | -23% | -12% | -16% |
| **Late** |  | -10% | -11% | -15% |  | 4% | 2% | 2% |  | 17% | 28% | 21% |
| **None** |  | -18% | -31% | -26% |  | 8% | 5% | 3% |  | 41% | 56% | 41% |
| **Advanced** |  | -4% | -4% | -4% |  | 0% | 1% | 0% |  | 3% | 6% | 5% |
